# Supplementary material for: Changing antibiotic prescribing practices in outpatient primary care settings in China: Study protocol for a health information system-based cluster-randomised crossover controlled trial
Source: PLoS One. 2022 Jan 7;17(1):e0259065. doi: 10.1371/journal.pone.0259065 (PMC8741015; doi:10.1371/journal.pone.0259065)
Supplement: S2 Appendix — (DOCX) [file pone.0259065.s002.docx]

Appendix

Deep learning and training of antibiotic prescription data

in Deep Graph Neural Network technology

In the artificial intelligence (AI) part of this study, the representation of relevant data and knowledge for training and model evaluation will first be addressed. Specifically, based on the results of big data analysis, the influencing factors of doctors and patients on the rational use of antibiotics will be summarized, and the Graph (Figure 1) model-based knowledge representation and modeling method will be studied in combination with the relevant contents of our self-made Guidance Recommendations on Clinical Use of Antibiotics in Primary Care Institutions.


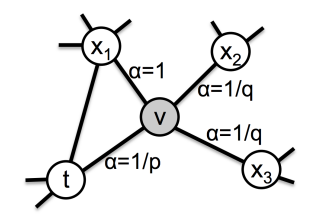


**Figure 1 Information Diagram with Edge**

After solving the representation problem of Graph model of training data, the Depth Graph Neural Network (DGNN) technology with Directed Graphs structure and edge-informative Graph structure will be studied (Figure 2). Specifically, a new heterogeneous and complex network structure model and iterative optimization method will be used. The DGNN method in the study makes use of several shallow network structures at the same time, with the depth of the traditional neural network dozens or even hundreds of layers in the stack to achieve higher network expression ability and performance. It can effectively avoid the traditional deep learning technology update iteration complex problem.


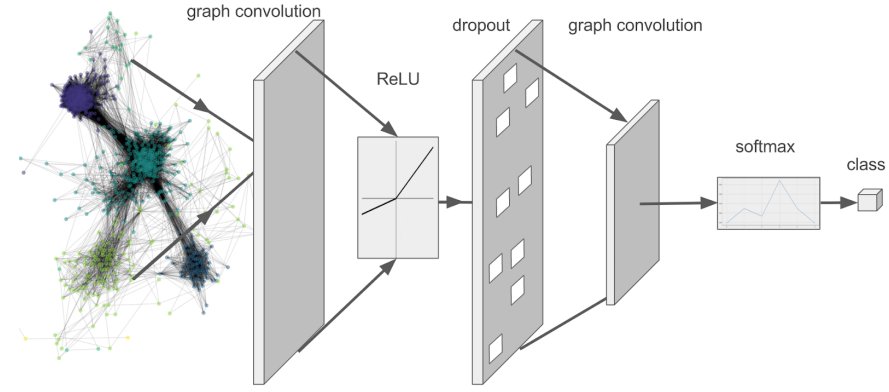


**Fig. 2 Schematic diagram of graph neural network with edge and direction information**

In order to improve the interpretability of the developed DGNN method in the process of antibiotic abuse assessment and analysis, the graph data representing antibiotic use path was visualized by similarity measurement and clustering technology based on graph data. Exploratory retrieval and presentation of multiple analysis results were provided to improve the comprehensibility and clinical reference value of the results of antibiotic prescription evaluation in this study.
